# HOME CARE NURSES CLAIM MEDICARE IGNORES SOCIAL DETERMINANTS OF HEALTH

**DOI:** 10.1093/geroni/igz038.554

**Published:** 2019-11-08

**Authors:** William D Cabin

**Affiliations:** Temple University, Philadelphia, Pennsylvania, United States

## Abstract

There is significant literature on the importance of addressing social determinants of health (SDOH) in order to improve health care outcomes. In response, the Centers for Medicare and Medicaid Services (CMS) has expanded Medicare Advantage plans ability to cover SDOH-related services. Medicare home health does not cover SDOH-related services. A literature review indicates no studies on the nature, significance, or impacts of the lack of SDOH coverage in Medicare home health. The current study is an initial, exploratory study to address the literature gap, based on interviews of a convenience sample of 37 home care nurses between January 2013 and May 2014 in the New York City metropolitan area. Results indicate nurses believe the lack of SDOH coverage in Medicare home health results in exacerbation of existing patient conditions; creation of new, additional patient conditions; increased home care readmissions and re-hospitalizations; increased caregiver burden; and exacerbation of patients’ mental health and substance abuse needs.

